# Capacity Building During Short-Term Surgical Outreach Trips: A Review of What Guidelines Exist

**DOI:** 10.1007/s00268-022-06760-1

**Published:** 2022-10-10

**Authors:** Chelsea Leversedge, Meghan McCullough, Luis Miguel Castro Appiani, Mùng Phan Đình, Robin N. Kamal, Lauren M. Shapiro

**Affiliations:** 1 VOICES Health Policy Research Center, Department of Orthopaedic Surgery, Stanford University, Redwood City, CA USA; 2 Department of Plastic Surgery, Stanford University, 450 Broadway Street, Redwood City, CA USA; 3 Department of Orthopaedic Surgery, Hospital Clinica Biblica, Aveinda 14 Calle 1 Y Central, San José, Costa Rica; 4 Orthropaedic Institute, 175 Military Hospital, Ho Chi Minh City, Vietnam; 5 VOICES Health Policy Research Center, Department of Orthopaedic Surgery, Stanford University, 450 Broadway Street MC: 6342, Redwood City, CA USA; 6 Department of Orthopaedic Surgery, University of California, 1500 Owens St., San Francisco, CA USA

## Abstract

**Introduction:**

While short-term surgical outreach trips improve access to care in low- and middle-income countries (LMIC), there is rising concern about their long-term impact. In response, many organizations seek to incorporate capacity building programs into their outreach efforts to help strengthen local health systems. Although leading organizations, like the World Health Organization (WHO), advocate for this approach, uniform guidelines are absent.

**Methods:**

We performed a systematic review, using search terms pertaining to capacity building guidelines during short-term surgical outreach trips. We extracted information on authorship, guideline development methodology, and guidelines relating to capacity building. Guidelines were classified according to the Global-QUEST framework, which outlines seven domains of capacity building on surgical outreach trips. Guideline development methodology frequencies and domain classifications frequencies were calculated; subsequently, guidelines were aggregated to develop a core guideline for each domain.

**Results:**

A total of 35 studies were included. Over 200 individual guidelines were extracted, spanning all seven framework domains. Guidelines were most frequently classified into Coordination and Community Impact domains and least frequently into the Finance domain. Less than half (46%) of studies collaborated with local communities to design the guidelines. Instead, guidelines were predominantly developed through author trip experience.

**Conclusion:**

As short-term surgical trips increase, further work is needed to standardize guidelines, create actionable steps, and promote collaborations in order to promote accountability during short-term surgical outreach trips.

## Introduction

Despite remarkable improvements in global health, global surgical care has historically been neglected. The field has gained more attention as the surgical global burden of disease surpasses that of HIV/AIDS, tuberculosis, and malaria combined [1, 2]. This burden is borne disproportionately by low- and middle-income countries (LMIC), where nine in ten individuals do not have access to surgical care and one in three patients reports negative experiences with their health system [1–4]. In response to this burden and with the purported growing evidence of the cost-effectiveness of short-term surgical outreach trips [5, 6], outreach trips are becoming increasingly common. Over 500 outreach organizations exist in the USA and an estimated 6000 trips are conducted annually [7, 8], typically aiming to increase access to much needed surgical services. However, the lasting impact of such trips has been criticized due to their short-term nature, perpetuation of voluntourism and colonialist practices, limited preparation and training in local care and customs, sparse outcome collection, and lack of regulation [8–12].

**Fig. 1:**
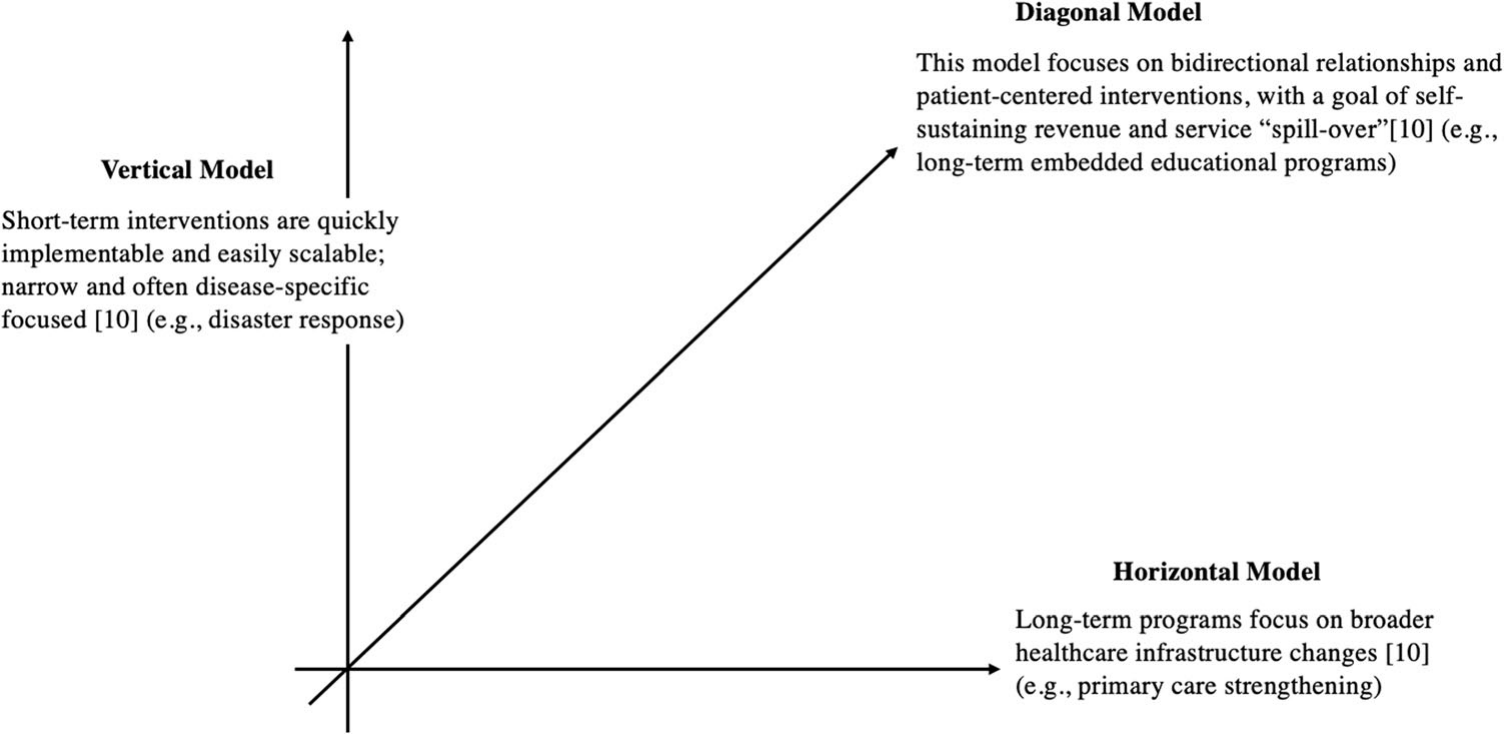
Outreach trip models

To counteract potential negative impacts, a paradigm shift toward a “diagonal” trip model is underway in response to the criticism of this traditionally “vertical approach,” which focuses on service delivery and specific diseases and typically operates outside the local healthcare system (Fig. 1) [13]. The diagonal model aims to integrate capacity building activities and programs into short-term surgical outreach trips by incorporating elements of “horizontal models,” which are more commonly long-term partnerships focusing on broadly strengthening the medical infrastructure [13]. Capacity building, as defined by the United Nations (UN), is the process of strengthening, adapting, and maintaining the ability to manage affairs successfully, over time [14]. In the context of surgical outreach trips, this includes strengthening personnel scope through educational and research opportunities, working toward local goals of self-sustaining revenue, and by enhancing infrastructure capabilities [13, 14].

Given these benefits, building surgical capacity in LMICs is a frequently cited goal by organizations such as the World Health Organization (WHO), several Lancet Commissions such as the Lancet Commission on Global Surgery [1, 4, 15], and by surgical outreach organizations [15–19]. It is also a commonly cited goal of local providers, who, when surveyed about their priorities within international collaborations, ranked professional development as the highest and direct care delivery as lowest priorities for international collaborations [20, 21].

Consensus regarding capacity building activities has been described for non-surgical outreach trips by DeCamp and colleagues, who outline seven core guidelines on ethics and clinical care [22]. The literature surrounding capacity building for surgical outreach trips, however, is primarily composed of anecdotal reports and recommendations. Efforts to better organize best practices have been recently undertaken in the hand surgery literature by Global Quality in Upper Extremity Surgery and Training (Global-QUEST) [23]. The purpose of their work is to guide capacity building activities for outreach organizations by outlining seven essential domains for capacity building [23]. The aim of this study is to evaluate guidelines and recommendations for capacity building during short-term surgical outreach trips and to evaluate them according to capacity building domains. In addition to presenting guidelines, this article aims to engage others conducting surgical outreach trips in local health system capacity building and to assist in guiding capacity building efforts.

## Methods

### Search strategy and selection criteria

We conducted a systematic review following the Preferred Reporting Items for Systematic Reviews and Meta-Analyses (PRISMA) guidelines to identify literature describing capacity building guidelines during short-term surgical outreach trips. We did not register the protocol publicly. We designed explicit search algorithms and queried PubMed, Web of Science, Embase, and ProQuest. Each database algorithm included “capacity building,” “guidelines,” and “outreach trips” along with their synonyms and database-specific search terms (Appendix 1). We reviewed the references of included studies [9] and carried out a review of the gray literature and of the WHO and Centers for Disease Control (CDC) databases using the same search terms.

There was no publication date or surgical sub-specialty restrictions. Exclusion criteria included trips without a surgical focus and long-term partnerships (defined as longer than eight weeks) [5]. All study types, except for systematic reviews and case studies, were eligible for inclusion. Inclusion criteria also included the presence of guidelines which, for the purpose of this study, are defined as a set of principles to follow, such as predefined, published guidelines, or best practice recommendations [9]. Only guidelines referring to capacity building activities during short-term surgical outreach trips as defined by the UN [14] were included, as opposed to clinical or resource-specific guidelines [9].

### Quality assessment and data collection

After screening, we performed a quality assessment of eligible articles using the Appraisal of Guidelines Research and Evaluation—Recommendations Excellence (AGREE-REX) tool. We used this tool to appraise each guideline by measuring quality within three domains: clinical applicability, values and preferences, and feasibility [24, 25]. Only studies with an overall score of high quality (> 70%) or of moderate quality (> 30%) were eligible for inclusion as recommended by the AGREE-REX tool [24].

We uploaded results onto Covidence, a Cochrane-sanctioned application for the screening and analysis of articles in systematic reviews. One author conducted the initial screening of titles and abstracts (CL). Two authors next conducted full text reviews of the remaining literature independently (CL and MM) and settled disputes during a research meeting, with a third author (LMS) available to settle discrepancies.

### Data extraction and analysis

We collected basic information about each included article, including authorship, guideline development methodology, any capacity building definition provided, and surgical specialty (if noted) of the trip. Each author list was analyzed for first author and then all author locations (or listed affiliations) to capture prevalence of LMIC authorship. We collected each paper’s methodology for guideline development and then grouped by methodology (e.g., guidelines created by author experience). We identified capacity building-related guidelines from each article and then classified each guideline by the Global-QUEST Capacity Building Framework and Operational Blueprint [23]. This framework includes seven domains to assess surgical capacity building activities, including: Partnership, Professional Development, Governance, Community Impact, Finance, Culture, and Coordination (outlined in Table 1). To ensure consistency in classification, two researchers (CL and MM) classified all guidelines from one manuscript as eligible or ineligible and then matched each to a domain together to ensure inter-coder reliability. In a second round of coding, the two authors classified each guideline from a second manuscript independently and then discussed differences during a research meeting. The remaining manuscripts were classified independently.

**Table 1:** Global-QUEST Capacity Domains

Domain	Definition
Partnership	Long-term connections, relationships, or their development between a surgical outreach organization, local healthcare system/community, and government
Professional development	The advancement of the clinical, educational, and research activities of the local healthcare system/community
Governance	The structures and processes by which a surgical outreach organization and local healthcare system/community are directed
Community impact	The effect of activities on the local community
Finance	The capital required to fund activities
Culture	The beliefs of the surgical outreach organization and the local healthcare system/community
Coordination	The communication and strategic planning for the execution of activities

After classifying all eligible guidelines into each domain, as seen in Table 1, we developed a core guideline from each domain. These are a combination of the most frequently noted guidelines and a compilation of common or similar themes [9].

## Results

**Fig. 2:**
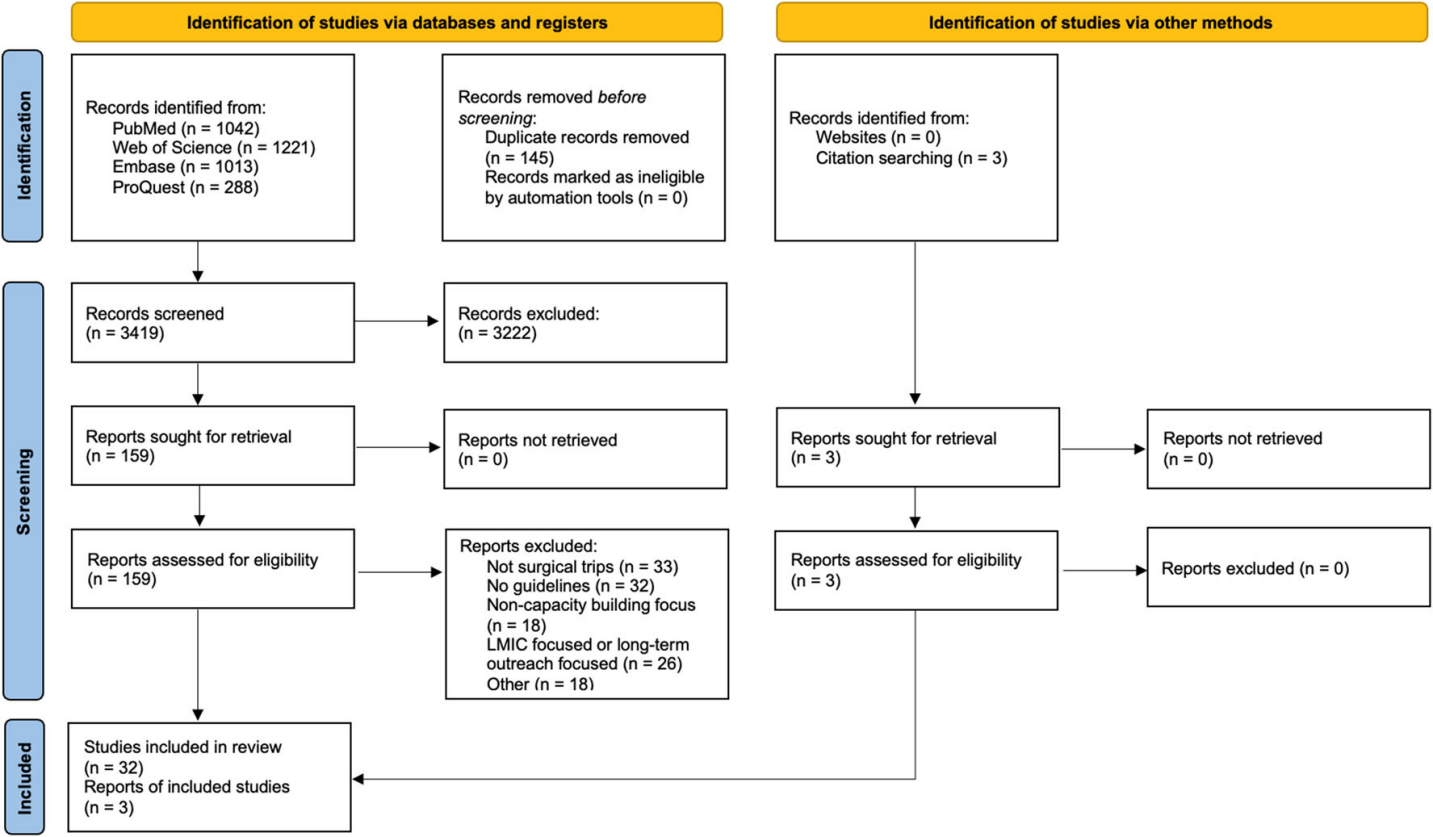
PRISMA 2020 Flow Diagram. *Figure legend.* From: Page MJ, McKenzie JE, Bossuyt PM, Boutron I, Hoffmann TC, Mulrow CD, et al. The PRISMA 2020 statement: an updated guideline for reporting systematic reviews. BMJ 2021; 372:n71. https://doi.org/10.1136/bmj.n71. For more information, visit: http://www.prisma-statement.org/

The initial search yielded 3564 articles; 145 duplicates were removed. In total, 35 articles were included (Fig. 2). All included articles met quality analysis thresholds.

### Level of guideline development

None of the first authors were from LMICs, but ten (29%) studies included authors from LMICs in their author list. Nearly half of the studies (16 studies, 44%) noted their guidelines, and best practices were created in collaboration with local stakeholders. When grouped by methodology (Table 2), the most common method of guideline design was through “expert consensus” (49%), such as through committee opinions or medical societies.

**Table 2:** Guideline development methodology

Guideline development methodology	Frequency [studies]
Expert consensus or collaborations	17 [28–42]
Author experience and perspective	8 [43–50]
Purposive sampling and opinion gathering	7 [8, 51–56]
Retrospective trip review	3 [57–59]

While the majority of studies included created their own guidelines in the absence of validated metrics, two studies evaluated the usability of the WHO Safe Surgery Checklist [28, 50] and one study mentioned WHO tools such as the WHO List of Priority Medical Devices for Cancer Management to address physical resource capacity [34].

The most frequently classified domain was Community Impact (from 23 (66%) studies) and the least frequent was Finance, with components from nine (25%) studies [28, 29, 35, 41, 48, 49, 55, 57, 58].

### Core guidelines

Extracted guidelines focusing on capacity building spanned all seven domains of the Global-QUEST framework, seen in Table 3. From each domain, the core guidelines are as follows:

1. The establishment of bidirectional and long-term **partnerships** with the local hospital, local organizations and government agencies, and community health workers is crucial for capacity building initiative implementation.
2. **Professional development** opportunities should be a focus of short-term surgical outreach trips, prioritizing sustained mentorship and peer education.
3. Surgical outreach organizations and volunteers must comply and cooperate with local **governance** and may not practice outside scope.
4. **Community impact** begins with a pre-trip needs assessment, includes continual outcome collection, and is contingent on successive trips to one location.
5. Flexible and reliable **finances** are required, with a goal of slowly tapering funding reliance to local hospitals as the results of capacity building initiatives replace the need for trips.
6. Respect for local **culture**, norms, and resources should be demonstrated throughout all components of trip planning, duration, and sustained partnerships.
7. **Coordination** of teams, continuity of care, and academic activities should be driven by local needs and priorities.

**Table 3:** Capacity building guideline elements by domain

Domain	Guideline elements	f	Sources
Partnerships	Establishment of bidirectional partnerships that are collaborative are critical	6	[29, 32, 41, 48, 52, 57]
	Short-term trips should be a component of long-term partnerships to mitigate adverse consequences, ensure partnerships do not end abruptly, and to ensure sustainability	5	[28, 29, 33, 41, 57]
	Engage with partners outside of the local hospital, such as public health officials, community health workers, local pharmacies, local NGOs, or local government agencies	11	[28, 33, 41, 46, 50, 53, 55–58]
	Foster coalitions between outreach organizations to streamline care	2	[42, 47]
Professional development	Education and skill development of local providers and volunteers should be integrated or focused on during trips	12	[8, 36, 37, 39, 42, 44, 47, 49, 50, 52, 53, 55]
	Any professional development initiatives should be adapted to local culture and context, with subject area identified by local health professionals	3	[33, 52, 54]
	Prioritize peer education and sustained mentorship over short lectures and do not allow HIC trainee education to overshadow those of local providers	6	[28, 29, 32, 36, 42, 58]
	Promote research capacity building and/or research collaborations	2	[49, 52]
	Expand local research infrastructure, and any publications or research should include co-authorship with involved local providers	3	[34, 36, 52]
	Explore new roles for local health providers (e.g., task shifting or sharing, Teach the Teacher)	5	[43, 49–51, 57]
Governance	Register and cooperate with local governance, including any required certifications from the host country and understanding local laws and regulations	7	[26, 28, 32, 33, 39, 43, 50, 54]
	Activities should align with the goals of the public sector, follow local patient protection practices, and meet local quality standards	6	[32, 33, 36, 48, 50, 56]
	Do not practice outside of scope, which is especially important when trainees (including students, residents, and fellows) are assisting during the trip	11	[26, 28–30, 33, 36, 39, 43, 50, 53]
	Follow guidelines for using any expired or reused supplies, leave any equipment necessary for follow-up care or future planned procedures with the local hospital	3	[28, 30, 32]
	Have policies in place for the informed consent and media collection process, including obtaining patient consent for any photography and research	5	[28, 33, 39, 42, 45]
Community impact	First priority is to do no harm	5	[31, 36, 42, 44, 47]
	Conduct a needs assessment before the trip to identify resources, local priorities, etc. and direct all trip activities by local community needs	11	[27–29, 38, 41–43, 46, 47, 50]
	Ensure appropriate outcomes, including measures of quality, are collected during trips and reviewed afterward	9	[8, 33, 35, 39, 41, 43, 45, 48, 50]
	Maintain an internal registry or database that is easily accessible and understood by all providers to collect patient outcomes	4	[39, 42, 47, 55]
	Use a safe surgery checklist during outreach	4	[32, 33, 35, 45]
	Ensure continuity of care by having a local clinician involved in all steps of care	3	[26, 33, 54]
	For long-term sustainability of impact, have successive trips in one location and focus on health systems strengthening to promote independence of health system	5	[8, 33, 48, 50, 58]
	Embed trips within community-led efforts and the local public health infrastructure	1	[48]
Finance	Monitor costs and ensure (organization) financial reports are easily accessible	3	[29, 35, 55]
	Explore fund allocation during trips, such as implementing a sliding payment scale to support patient care and allocate funds to more sustainable health projects	4	[28, 41, 48, 49]
	Have reliable and flexible funding that gradually decreases over time as care becomes sustainable	2	[57, 58]
Culture	Volunteers should be familiar with and demonstrate respect for the local community’s culture, norms, challenges, and constraints	8	[26–28, 30, 31, 36, 48, 52]
	Pre-departure preparation in cultural barriers, cross-cultural effectiveness, cultural sensitivity, and cultural humility for volunteers is necessary	5	[36, 39, 40, 48, 55]
	All materials (e.g., consent) should be adapted to local standards, language, and situation (access to resources, infrastructure, etc.)	1	[29, 33]
	Ensure patient consent, education, and shared decision making occurs without coercion, with respect for language and cultural factors, and with a translator present	4	[30, 32, 33, 36, 39, 53]
Coordination	Have a clear goal and expectations for the trip, all of which are based on local priorities and constraints	6	[29, 40, 41, 47, 53, 58]
	There is an established communication system between local physicians and the organization	1	[39]
	Pre-trip planning should include communication of pre-selected patients (if desired), the infrastructure to provide care, risk factors, and conditions needed to treat	6	[28, 30, 34, 36, 37, 39, 59]
	Collaborations with other organizations, such as local NGOs or other international outreach organizations, is recommended	4	[30, 34, 43, 44]
	The volunteer team has a formal staffing plan and should be multidisciplinary	6	[41, 43, 47, 50, 55]
	Ensure there is a plan for referrals and follow-up	10	[28, 30, 36, 38, 39, 41, 47, 50, 55]
	Have post-trip debriefing and solicit feedback from multiple stakeholders to reflect and plan next trips	3	[29, 36, 47]

## Discussion

Concerns regarding the impact of short-term surgical outreach trips have prompted discussions about, and inclusion of, capacity building initiatives as part of outreach trips. The traditional vertical approach to surgical aid has been criticized for a host of shortcomings, not only related to discontinuity of care for patients and a lack of emphasis on the development of local providers and health systems, but for perpetuation of power imbalances between LMIC and HICs and undertones of neocolonialism. While many leading organizations advocate for the incorporation of capacity building as part of a shift toward a more diagonal approach, there is a lack of consensus guidelines for how best to carry this out and how to move past the constraints of short-term surgical aid. In this review, we identified primarily manuscripts with descriptive or anecdotal recommendations and consolidated the recommendations into capacity building domains. These recommendations were found to span all seven domains of the applied capacity building framework, but there is a further need to ensure standardization and to promote accountability during short-term surgical outreach trips.

In our analysis we found a lack of representation of LMIC voices guiding these discussions despite the known importance of cultural and setting-specific contextualization. Although many recommendations identified in this study noted the importance of inclusion of local leaders and personnel in capacity building activities and trip planning, only about half of the manuscripts captured by this study included authors from LMICs in their author list. This illustrates the concern of “parachute” or “helicopter research” dominating capacity building guidelines for LMICs [60, 61]. Such a research approach includes publishing findings about primary research in countries yet neglecting to recognize local researchers [60, 61]. Unequal research opportunities or minimal collaboration perpetuates power imbalances, visible in this study as well as in a recent analysis that found 30% of research publications conducted in LMICs did not include any local authors [9, 60, 62]. In contrast, collaboration in research establishes mutually beneficial relationships, adapts guidelines and findings to cultural contexts, empowers local communities, and minimizes potential burdens put on local staff or resources during trips [7, 22]. There is emerging literature on potential ways to increase engagement of authorship from LMICs, with a recent consensus statement on this challenge from leading editors from global health journals such as Morton and colleagues [60]. Some suggestions posed by this expert panel, such as inclusion of reflexivity statements when submitting research conducted in LMICs and removing journal authorship limits, may be aspects to address [60].

Our analysis further supports the importance of local leadership in the development of guidelines and an awareness and respect for cultural contextualization. Additionally, there were no manuscripts which field tested or validated their guidelines. Current recommendations for guideline development and consensus building, as outlined by the WHO, suggest the development of a systematic search for evidence, inclusion of external reviews, and incorporation of community attributes [63–65]. Adaptation of guidelines should also include an analysis or study of the validation and feasibility of guidelines through implementation science, especially when working on a global scale or when working in contexts that are different from where they are being developed [66–68]. By utilizing an implementation science or a field-testing approach, factors such as cultural context and health system differences can be integrated to allow for guideline correction and a positive impact in alignment with local context, culture, and health system priorities [66].

When examining which capacity building domains were represented, culture and community domains were identified in nearly all guidelines. This finding is reinforced by a number of recent surveys of providers during outreach trips, where local and visiting care team providers most commonly highlighted knowledge of local culture as a necessary skill for visiting providers [10, 69]. Preparing visiting providers for unfamiliar contexts, including language, dress standards, and the kind of patients to expect was noted as vitally important [10]. Many groups have recently used the Intercultural Effectiveness Scale (IES) survey to address a lack of understanding in these areas. This scale ranks cultural competence on a scale of 1 to 7 and can be used to develop pre-trip cultural preparation [70, 71]. Using tools such as the IES or including cultural competence preparation is important for professional development, as a lack of this preparation is likely to make professional development programming, or any other capacity building program, less impactful [48].

Guideline components in the finance domain were least commonly published, with only nine (25%) noting the importance of exploring new funding models to promote local health system revenue independence. This represents an area for research and guideline creation, especially as recent literature suggests that funding surgical scale-up, while initially a larger financial investment, is ultimately cost-effective [1]. Investing financially in local surgical infrastructure is important as surgical diseases result in up to 2.5% loss of potential gross domestic product (GDP) for LMICs along with substantial health losses [1, 72]. Some outreach trips have proposed different methods of building financial capacity in surgical systems to assist in bridging the initial high cost of surgical scale-up, such as a sliding scale patient payment system to financially support trips and allow for funds to be allocated to larger health projects [48] or calculating and reimbursing all costs to the local community during the trip [22]. We recommend continued investigation into and incorporation of sustainable financial programs and models during short-term surgical outreach trips.

While many efforts to include capacity building during outreach trips were present in this study, actionable steps were noticeably missing. The majority of guidelines were presented descriptively as best practices, with two guidelines presented as manuals or checklists [33, 39]. Importantly, we found no incentivizing mechanisms for adoption of capacity building during short-term surgical outreach trips and no research detailing guideline enforcement. While there is no method to quantify how many organizations are following or using such guidelines, previous studies have found low implementation and use of guidelines and evaluation of practices during short-term outreach trips [5, 9, 10, 53]. Whether this is due to usability, applicability, or other factors, further work is needed to ensure guidelines are actionable and to understand how to better integrate capacity building practices into short-term surgical outreach.

The findings of this study should be viewed in light of their limitations. As a review of current capacity building guidelines, we did not pilot test the guidelines presented. Furthermore, there may be a publication bias present. Guidelines and recommendations presented here may not capture all guidelines used or followed by outreach organizations, and the studies included represent a small proportion of trips conducted worldwide [73]. Further, there may be trips reporting outcomes or publishing studies that do not report which guidelines they followed.

## Conclusion

In conclusion, current guidelines for surgical outreach trips span all domains of capacity building, but there is a discrepancy in frequency of domain representation, guidelines are primarily anecdotal in nature, and there is limited input from LMIC authors and communities. As surgical outreach trips continue to increase, further work is needed to create standardized, actionable, and measurable guidelines in order to promote and unify best capacity building practices during these trips.

## Appendix 1

Search Strategy.

### PubMed

((“Capacity building”[mesh] OR “Quality improvement”[mesh] OR “best practices”[tw]) AND (medical missions[mesh] OR "surgical mission*"[tw] OR "religious missions"[mesh] OR "outreach trip*"[tiab] OR "global outreach*"[tw] OR “global health”[tw] OR “global health”[mesh] OR "international outreach"[tw] OR humanitarian*[tw] OR "short term experience"[tw] OR “developing countries”[mesh] OR “volunteer*”[tw]) AND (“delivery of health care”[mesh] OR “guidelines as topic”[mesh] OR “priorit*”[tw] OR “needs assessment”[mesh] OR “guideline*”[tw] OR “evidence-based practice”[mesh])).

### Web of science

1. TS = (capacity building) OR TS = (quality improvement)
2. TS = (medical mission) OR TS = (humanitarian aid) OR TS = (global surgery) OR TS = (surgical mission)
3. #1 AND #2
4. TS = (guideline*) OR TS = (best practice) OR TS = (needs assessment) OR TS = (standard*)
5. #3 AND #4

### Embase

1. '**capacity building**'/exp OR **'health care quality**'/exp
2. **'humanitarian aid'**/exp OR **'global health'**/exp OR **'medical mission*'**:ab,ti OR **'surgical mission*'**:ab,ti OR **'global surger*'**:ab,ti OR **'medical service trip*'**:ab,ti OR **'short term medical mission'**
3. **#1 AND #2**
4. **'guideline'**/exp OR **'practice guideline'**/exp OR **'standards'**/exp OR **'needs assessment'**/exp OR **'best practices’** OR ‘**evidence based medicine’**/exp
5. **#3 AND #4**

Email alert set.

### ProQuest

("medical mission" OR "global outreach" OR "surgical outreach" OR "international outreach") AND ("capacity building" or "quality improvement") AND (“guideline*” OR “needs assessment” OR “best practices”).
